# 2-Bromo-1,3-diphenyl­propan-1,3-dione

**DOI:** 10.1107/S1600536808038646

**Published:** 2008-11-26

**Authors:** Zainudin Arifin, Seik Weng Ng

**Affiliations:** a Department of Chemistry, University of Malaya, 50603 Kuala Lumpur, Malaysia

## Abstract

The title compound, C_15_H_11_BrO_2_, exists as a diketone in which the two benzoyl groups are nearly perpendicular to each other [dihedral angles = 79.9 (1) and 87.4 (1)° in the two independent mol­ecules].

## Related literature

The compound is claimed to exist in the enol form as it condenses with 2-amino­thia­zole and 2-mercaptoimidazoline; see: Robert & Panouse (1979 ▶). The parent dibenzoyl­methane mol­ecule exists in two modications, as 1,3-diphenyl-1-hydroxy­propen-1-one; see: Kaitner & Meštrović (1993 ▶); Ozturk *et al.* (1997 ▶).
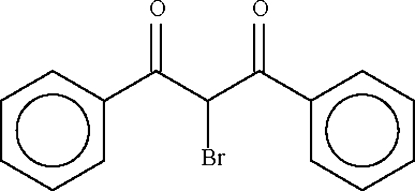


## Experimental

### 

#### Crystal data

C_15_H_11_BrO_2_
*M* *_r_* = 303.15
Orthorhombic, 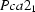

*a* = 28.0680 (6) Å
*b* = 5.6508 (1) Å
*c* = 15.3741 (3) Å
*V* = 2438.43 (8) Å^3^
*Z* = 8
Mo *K*α radiation
μ = 3.36 mm^−1^
*T* = 100 (2) K
0.27 × 0.20 × 0.06 mm

#### Data collection

Bruker SMART APEX diffractometer
Absorption correction: multi-scan (*SADABS*; Sheldrick, 1996 ▶) *T*_min_ = 0.464, *T*_max_ = 0.824
21610 measured reflections
5562 independent reflections
4774 reflections with *I* > 2σ(*I*)
*R*_int_ = 0.060

#### Refinement

*R*[*F* ^2^ > 2σ(*F* ^2^)] = 0.037
*wR*(*F* ^2^) = 0.086
*S* = 0.99
5562 reflections
325 parameters
1 restraint
H-atom parameters constrained
Δρ_max_ = 0.76 e Å^−3^
Δρ_min_ = −0.52 e Å^−3^
Absolute structure: Flack (1983 ▶), 2644 Fridel pairs
Flack parameter: −0.002 (9)

### 

Data collection: *APEX2* (Bruker, 2007 ▶); cell refinement: *SAINT* (Bruker, 2007 ▶); data reduction: *SAINT*; program(s) used to solve structure: *SHELXS97* (Sheldrick, 2008 ▶); program(s) used to refine structure: *SHELXL97* (Sheldrick, 2008 ▶); molecular graphics: *X-SEED* (Barbour, 2001 ▶); software used to prepare material for publication: *publCIF* (Westrip, 2008 ▶).

## Supplementary Material

Crystal structure: contains datablocks global, I. DOI: 10.1107/S1600536808038646/pk2135sup1.cif

Structure factors: contains datablocks I. DOI: 10.1107/S1600536808038646/pk2135Isup2.hkl

Additional supplementary materials:  crystallographic information; 3D view; checkCIF report

## Figures and Tables

**Fig. 1.**
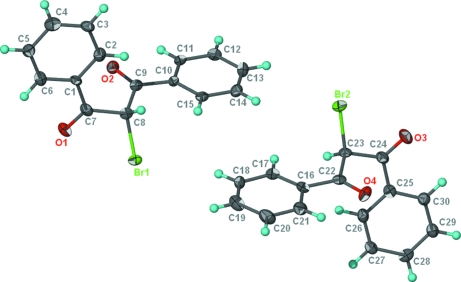
Thermal ellipsoid (Barbour, 2001) plot of the two independent molecules of C15H11BrO2 at the 70% probability level. Hydrogen atoms are drawn as spheres of arbitrary radius.

## supplementary crystallographic information

### Experimental

The commerically available compound was recrystallized from toluene.

### Refinement

Carbon-bound H-atoms were placed in calculated positions (C—H 0.95 Å) and were included in the refinement using a riding model approximation, with *U*(H) set to 1.2*U*(C).

The (200) reflection was omitted.

### Crystal data

**Table:**

C_15_H_11_BrO_2_	*F*_000_ = 1216
*M**_r_* = 303.15	*D*_x_ = 1.652 Mg m^−^^3^
Orthorhombic, *P**c**a*2_1_	Mo *K*α radiation λ = 0.71073 Å
Hall symbol: P 2c -2ac	Cell parameters from 4583 reflections
*a* = 28.0680 (6) Å	θ = 2.6–25.4º
*b* = 5.6508 (1) Å	µ = 3.36 mm^−^^1^
*c* = 15.3741 (3) Å	*T* = 100 (2) K
*V* = 2438.43 (8) Å^3^	Plate, colorless
*Z* = 8	0.27 × 0.20 × 0.06 mm

### Data collection

**Table:**

Bruker SMART APEX diffractometer	5562 independent reflections
Radiation source: fine-focus sealed tube	4774 reflections with *I* > 2σ(*I*)
Monochromator: graphite	*R*_int_ = 0.060
*T* = 173(2) K	θ_max_ = 27.5º
ω scans	θ_min_ = 2.0º
Absorption correction: Multi-scan(SADABS; Sheldrick, 1996)	*h* = −36→35
*T*_min_ = 0.464, *T*_max_ = 0.824	*k* = −7→7
21610 measured reflections	*l* = −19→19

### Refinement

**Table:**

Refinement on *F*^2^	Hydrogen site location: inferred from neighbouring sites
Least-squares matrix: full	H-atom parameters constrained
*R*[*F*^2^ > 2σ(*F*^2^)] = 0.037	*w* = 1/[σ^2^(*F*_o_^2^) + (0.0511*P*)^2^] where *P* = (*F*_o_^2^ + 2*F*_c_^2^)/3
*wR*(*F*^2^) = 0.087	(Δ/σ)_max_ = 0.001
*S* = 0.99	Δρ_max_ = 0.76 e Å^−^^3^
5562 reflections	Δρ_min_ = −0.52 e Å^−^^3^
325 parameters	Extinction correction: none
1 restraint	Absolute structure: Flack (1983), 2644 Fridel pairs
Primary atom site location: structure-invariant direct methods	Flack parameter: −0.002 (9)
Secondary atom site location: difference Fourier map	

### Fractional atomic coordinates and isotropic or equivalent isotropic displacement parameters (Å^2^)

**Table:**

	*x*	*y*	*z*	*U*_iso_*/*U*_eq_	
Br1	0.264659 (13)	0.21340 (7)	0.50000 (3)	0.01660 (9)	
Br2	0.355868 (14)	1.36089 (7)	0.23689 (3)	0.01981 (10)	
O1	0.18018 (11)	−0.0909 (5)	0.52553 (18)	0.0212 (7)	
O2	0.17207 (11)	0.1525 (5)	0.33248 (18)	0.0181 (6)	
O3	0.44667 (12)	1.6039 (6)	0.1914 (2)	0.0312 (8)	
O4	0.44450 (11)	1.4517 (5)	0.39763 (19)	0.0216 (7)	
C1	0.11351 (15)	0.1585 (6)	0.5074 (3)	0.0147 (8)	
C2	0.09476 (15)	0.3667 (8)	0.4735 (3)	0.0183 (9)	
H2	0.1153	0.4846	0.4505	0.022*	
C3	0.04576 (15)	0.4012 (8)	0.4733 (3)	0.0188 (9)	
H3	0.0330	0.5411	0.4482	0.023*	
C4	0.01566 (16)	0.2367 (7)	0.5086 (3)	0.0233 (10)	
H4	−0.0178	0.2623	0.5080	0.028*	
C5	0.03430 (16)	0.0314 (8)	0.5455 (3)	0.0208 (9)	
H5	0.0136	−0.0818	0.5711	0.025*	
C6	0.08317 (16)	−0.0077 (8)	0.5446 (3)	0.0194 (9)	
H6	0.0959	−0.1481	0.5695	0.023*	
C7	0.16522 (15)	0.1006 (7)	0.5035 (3)	0.0171 (8)	
C8	0.19934 (14)	0.2929 (7)	0.4686 (3)	0.0145 (8)	
H8	0.1906	0.4505	0.4936	0.017*	
C9	0.19594 (15)	0.3008 (7)	0.3688 (3)	0.0150 (8)	
C10	0.22242 (14)	0.4836 (7)	0.3189 (2)	0.0138 (8)	
C11	0.22560 (13)	0.4551 (7)	0.2283 (3)	0.0165 (8)	
H11	0.2121	0.3196	0.2013	0.020*	
C12	0.24831 (16)	0.6234 (8)	0.1785 (3)	0.0168 (9)	
H12	0.2501	0.6042	0.1172	0.020*	
C13	0.26858 (15)	0.8212 (8)	0.2174 (3)	0.0188 (9)	
H13	0.2841	0.9373	0.1829	0.023*	
C14	0.26596 (15)	0.8482 (7)	0.3067 (3)	0.0176 (9)	
H14	0.2800	0.9830	0.3334	0.021*	
C15	0.24308 (15)	0.6804 (7)	0.3579 (3)	0.0153 (8)	
H15	0.2416	0.7000	0.4192	0.018*	
C16	0.39938 (16)	1.1076 (8)	0.4260 (3)	0.0168 (9)	
C17	0.37847 (15)	0.9021 (8)	0.3946 (3)	0.0174 (9)	
H17	0.3785	0.8690	0.3341	0.021*	
C18	0.35758 (18)	0.7456 (8)	0.4527 (3)	0.0198 (10)	
H18	0.3440	0.6026	0.4318	0.024*	
C19	0.3564 (2)	0.7951 (9)	0.5399 (3)	0.0252 (11)	
H19	0.3413	0.6878	0.5786	0.030*	
C20	0.37725 (19)	1.0017 (9)	0.5725 (3)	0.0272 (11)	
H20	0.3769	1.0341	0.6331	0.033*	
C21	0.39847 (16)	1.1585 (7)	0.5150 (3)	0.0214 (10)	
H21	0.4124	1.3006	0.5361	0.026*	
C22	0.42297 (14)	1.2851 (8)	0.3686 (3)	0.0159 (8)	
C23	0.42002 (14)	1.2619 (8)	0.2686 (3)	0.0159 (9)	
H23	0.4256	1.0942	0.2505	0.019*	
C24	0.45729 (14)	1.4232 (7)	0.2285 (3)	0.0187 (8)	
C25	0.50847 (14)	1.3574 (7)	0.2417 (3)	0.0163 (8)	
C26	0.52174 (17)	1.1460 (7)	0.2826 (3)	0.0175 (9)	
H26	0.4980	1.0340	0.2985	0.021*	
C27	0.56937 (15)	1.0990 (7)	0.3001 (3)	0.0170 (9)	
H27	0.5783	0.9553	0.3276	0.020*	
C28	0.60386 (16)	1.2646 (8)	0.2768 (3)	0.0193 (9)	
H28	0.6364	1.2366	0.2901	0.023*	
C29	0.59053 (14)	1.4708 (7)	0.2339 (3)	0.0190 (8)	
H29	0.6141	1.5809	0.2159	0.023*	
C30	0.54305 (15)	1.5154 (8)	0.2177 (2)	0.0179 (9)	
H30	0.5342	1.6581	0.1893	0.022*	

### Atomic displacement parameters (Å^2^)

**Table:**

	*U*^11^	*U*^22^	*U*^33^	*U*^12^	*U*^13^	*U*^23^
Br1	0.01624 (19)	0.01938 (19)	0.01417 (16)	0.00186 (15)	−0.00319 (19)	−0.00273 (19)
Br2	0.01400 (19)	0.0249 (2)	0.02058 (18)	0.00115 (17)	−0.0026 (2)	−0.00051 (18)
O1	0.0241 (17)	0.0178 (16)	0.0216 (15)	0.0040 (13)	0.0017 (12)	0.0053 (11)
O2	0.0206 (17)	0.0167 (16)	0.0169 (14)	−0.0018 (12)	−0.0004 (12)	−0.0016 (12)
O3	0.0200 (18)	0.026 (2)	0.047 (2)	0.0031 (14)	−0.0006 (16)	0.0203 (16)
O4	0.0191 (17)	0.0190 (16)	0.0269 (16)	−0.0045 (13)	0.0007 (13)	−0.0065 (13)
C1	0.018 (2)	0.015 (2)	0.0107 (18)	0.0009 (14)	−0.0032 (17)	0.0021 (18)
C2	0.023 (2)	0.016 (2)	0.0162 (19)	−0.0028 (17)	0.0000 (17)	−0.0040 (15)
C3	0.020 (2)	0.016 (2)	0.021 (2)	0.0056 (16)	−0.0044 (17)	0.0010 (16)
C4	0.017 (2)	0.027 (3)	0.025 (2)	0.0053 (16)	0.001 (2)	−0.002 (2)
C5	0.020 (2)	0.027 (3)	0.015 (2)	−0.0058 (19)	0.0001 (17)	0.0050 (18)
C6	0.023 (2)	0.017 (2)	0.018 (2)	−0.0011 (17)	0.0024 (18)	0.0005 (17)
C7	0.024 (2)	0.018 (2)	0.0094 (16)	0.0007 (16)	0.0012 (18)	0.0010 (19)
C8	0.015 (2)	0.015 (2)	0.0140 (16)	0.0032 (16)	0.0006 (15)	−0.0054 (16)
C9	0.016 (2)	0.013 (2)	0.0155 (18)	0.0025 (17)	−0.0016 (16)	−0.0046 (15)
C10	0.011 (2)	0.014 (2)	0.0168 (19)	−0.0004 (15)	−0.0006 (15)	0.0013 (16)
C11	0.0124 (18)	0.018 (2)	0.019 (2)	−0.0006 (15)	−0.0032 (19)	0.0013 (18)
C12	0.022 (2)	0.021 (2)	0.0079 (17)	0.0013 (17)	−0.0003 (16)	−0.0039 (17)
C13	0.020 (2)	0.016 (2)	0.021 (2)	−0.0026 (16)	0.0036 (17)	−0.0006 (16)
C14	0.018 (2)	0.015 (2)	0.021 (2)	−0.0008 (17)	−0.0016 (17)	−0.0037 (16)
C15	0.016 (2)	0.016 (2)	0.0138 (18)	0.0020 (16)	−0.0038 (16)	−0.0024 (16)
C16	0.018 (2)	0.017 (2)	0.0153 (19)	0.0037 (17)	−0.0010 (17)	0.0029 (17)
C17	0.016 (2)	0.021 (2)	0.0158 (19)	0.0024 (17)	−0.0009 (17)	0.0000 (17)
C18	0.014 (2)	0.017 (2)	0.028 (3)	−0.0018 (18)	0.000 (2)	0.0058 (18)
C19	0.024 (3)	0.026 (3)	0.026 (2)	0.006 (2)	0.007 (2)	0.006 (2)
C20	0.038 (3)	0.029 (3)	0.014 (2)	0.002 (2)	−0.0012 (19)	0.0036 (18)
C21	0.026 (2)	0.017 (2)	0.021 (2)	0.0014 (17)	−0.0040 (18)	−0.0013 (17)
C22	0.010 (2)	0.019 (2)	0.0186 (19)	0.0059 (17)	−0.0013 (16)	−0.0006 (17)
C23	0.008 (2)	0.021 (2)	0.0192 (19)	0.0036 (16)	−0.0002 (16)	0.0040 (16)
C24	0.020 (2)	0.020 (2)	0.016 (2)	0.0006 (16)	0.000 (2)	0.0043 (18)
C25	0.018 (2)	0.0157 (19)	0.0155 (18)	0.0032 (17)	0.004 (2)	−0.0018 (17)
C26	0.024 (2)	0.012 (2)	0.017 (2)	−0.0009 (17)	0.0009 (18)	−0.0021 (16)
C27	0.022 (2)	0.013 (2)	0.0164 (19)	0.0042 (16)	0.0014 (17)	−0.0012 (15)
C28	0.016 (2)	0.023 (2)	0.019 (2)	0.0013 (17)	0.0012 (17)	−0.0073 (18)
C29	0.021 (2)	0.018 (2)	0.0180 (17)	−0.0010 (16)	0.003 (2)	0.0013 (19)
C30	0.018 (2)	0.017 (2)	0.020 (2)	0.0005 (16)	0.0001 (16)	0.0003 (16)

### Geometric parameters (Å, °)

**Table:**

Br1—C8	1.948 (4)	C14—C15	1.389 (6)
Br2—C23	1.947 (4)	C14—H14	0.9500
O1—C7	1.209 (5)	C15—H15	0.9500
O2—C9	1.209 (5)	C16—C17	1.388 (6)
O3—C24	1.207 (5)	C16—C21	1.398 (6)
O4—C22	1.204 (5)	C16—C22	1.491 (6)
C1—C2	1.390 (6)	C17—C18	1.387 (6)
C1—C6	1.391 (6)	C17—H17	0.9500
C1—C7	1.489 (6)	C18—C19	1.369 (6)
C2—C3	1.389 (6)	C18—H18	0.9500
C2—H2	0.9500	C19—C20	1.399 (7)
C3—C4	1.369 (6)	C19—H19	0.9500
C3—H3	0.9500	C20—C21	1.386 (6)
C4—C5	1.393 (6)	C20—H20	0.9500
C4—H4	0.9500	C21—H21	0.9500
C5—C6	1.389 (6)	C22—C23	1.545 (6)
C5—H5	0.9500	C23—C24	1.518 (6)
C6—H6	0.9500	C23—H23	1.0000
C7—C8	1.544 (6)	C24—C25	1.498 (6)
C8—C9	1.538 (5)	C25—C30	1.370 (6)
C8—H8	1.0000	C25—C26	1.400 (6)
C9—C10	1.487 (6)	C26—C27	1.389 (6)
C10—C15	1.391 (6)	C26—H26	0.9500
C10—C11	1.405 (6)	C27—C28	1.393 (6)
C11—C12	1.377 (6)	C27—H27	0.9500
C11—H11	0.9500	C28—C29	1.390 (6)
C12—C13	1.390 (6)	C28—H28	0.9500
C12—H12	0.9500	C29—C30	1.379 (6)
C13—C14	1.384 (6)	C29—H29	0.9500
C13—H13	0.9500	C30—H30	0.9500
			
C2—C1—C6	119.6 (4)	C17—C16—C21	120.3 (4)
C2—C1—C7	122.7 (4)	C17—C16—C22	123.0 (4)
C6—C1—C7	117.7 (3)	C21—C16—C22	116.7 (4)
C3—C2—C1	119.7 (4)	C18—C17—C16	119.3 (4)
C3—C2—H2	120.2	C18—C17—H17	120.4
C1—C2—H2	120.2	C16—C17—H17	120.4
C4—C3—C2	121.0 (4)	C19—C18—C17	120.7 (5)
C4—C3—H3	119.5	C19—C18—H18	119.7
C2—C3—H3	119.5	C17—C18—H18	119.7
C3—C4—C5	119.7 (4)	C18—C19—C20	120.7 (5)
C3—C4—H4	120.2	C18—C19—H19	119.6
C5—C4—H4	120.2	C20—C19—H19	119.6
C6—C5—C4	120.0 (4)	C21—C20—C19	119.0 (4)
C6—C5—H5	120.0	C21—C20—H20	120.5
C4—C5—H5	120.0	C19—C20—H20	120.5
C5—C6—C1	120.0 (4)	C20—C21—C16	120.0 (4)
C5—C6—H6	120.0	C20—C21—H21	120.0
C1—C6—H6	120.0	C16—C21—H21	120.0
O1—C7—C1	121.6 (4)	O4—C22—C16	121.9 (4)
O1—C7—C8	120.8 (4)	O4—C22—C23	117.5 (4)
C1—C7—C8	117.6 (3)	C16—C22—C23	120.5 (4)
C9—C8—C7	109.2 (3)	C24—C23—C22	108.4 (3)
C9—C8—Br1	108.2 (3)	C24—C23—Br2	111.3 (3)
C7—C8—Br1	109.6 (3)	C22—C23—Br2	105.9 (3)
C9—C8—H8	110.0	C24—C23—H23	110.4
C7—C8—H8	110.0	C22—C23—H23	110.4
Br1—C8—H8	110.0	Br2—C23—H23	110.4
O2—C9—C10	121.3 (4)	O3—C24—C25	120.7 (4)
O2—C9—C8	118.4 (4)	O3—C24—C23	122.0 (4)
C10—C9—C8	120.3 (3)	C25—C24—C23	117.2 (3)
C15—C10—C11	119.5 (4)	C30—C25—C26	119.3 (4)
C15—C10—C9	122.8 (4)	C30—C25—C24	118.8 (4)
C11—C10—C9	117.7 (3)	C26—C25—C24	121.8 (4)
C12—C11—C10	120.1 (4)	C27—C26—C25	120.4 (4)
C12—C11—H11	120.0	C27—C26—H26	119.8
C10—C11—H11	120.0	C25—C26—H26	119.8
C11—C12—C13	120.4 (4)	C26—C27—C28	119.4 (4)
C11—C12—H12	119.8	C26—C27—H27	120.3
C13—C12—H12	119.8	C28—C27—H27	120.3
C14—C13—C12	119.6 (4)	C29—C28—C27	119.9 (4)
C14—C13—H13	120.2	C29—C28—H28	120.1
C12—C13—H13	120.2	C27—C28—H28	120.1
C13—C14—C15	120.8 (4)	C30—C29—C28	119.9 (4)
C13—C14—H14	119.6	C30—C29—H29	120.0
C15—C14—H14	119.6	C28—C29—H29	120.0
C14—C15—C10	119.6 (4)	C25—C30—C29	121.1 (4)
C14—C15—H15	120.2	C25—C30—H30	119.4
C10—C15—H15	120.2	C29—C30—H30	119.4
			
C6—C1—C2—C3	−3.2 (6)	C21—C16—C17—C18	−1.4 (7)
C7—C1—C2—C3	175.0 (4)	C22—C16—C17—C18	179.5 (4)
C1—C2—C3—C4	2.2 (6)	C16—C17—C18—C19	1.7 (8)
C2—C3—C4—C5	0.1 (7)	C17—C18—C19—C20	−1.5 (9)
C3—C4—C5—C6	−1.3 (7)	C18—C19—C20—C21	1.1 (8)
C4—C5—C6—C1	0.3 (6)	C19—C20—C21—C16	−0.8 (7)
C2—C1—C6—C5	1.9 (6)	C17—C16—C21—C20	1.0 (7)
C7—C1—C6—C5	−176.4 (4)	C22—C16—C21—C20	−179.9 (4)
C2—C1—C7—O1	−172.5 (4)	C17—C16—C22—O4	−173.4 (4)
C6—C1—C7—O1	5.7 (6)	C21—C16—C22—O4	7.5 (6)
C2—C1—C7—C8	6.3 (6)	C17—C16—C22—C23	7.3 (6)
C6—C1—C7—C8	−175.4 (4)	C21—C16—C22—C23	−171.9 (4)
O1—C7—C8—C9	101.3 (5)	O4—C22—C23—C24	15.6 (5)
C1—C7—C8—C9	−77.6 (5)	C16—C22—C23—C24	−165.0 (4)
O1—C7—C8—Br1	−17.1 (5)	O4—C22—C23—Br2	−103.9 (4)
C1—C7—C8—Br1	164.1 (3)	C16—C22—C23—Br2	75.4 (4)
C7—C8—C9—O2	−6.1 (5)	C22—C23—C24—O3	−107.7 (5)
Br1—C8—C9—O2	113.1 (4)	Br2—C23—C24—O3	8.4 (6)
C7—C8—C9—C10	175.3 (3)	C22—C23—C24—C25	68.5 (5)
Br1—C8—C9—C10	−65.5 (4)	Br2—C23—C24—C25	−175.4 (3)
O2—C9—C10—C15	168.2 (4)	O3—C24—C25—C30	5.6 (7)
C8—C9—C10—C15	−13.3 (6)	C23—C24—C25—C30	−170.6 (4)
O2—C9—C10—C11	−10.7 (6)	O3—C24—C25—C26	−178.3 (4)
C8—C9—C10—C11	167.9 (4)	C23—C24—C25—C26	5.5 (6)
C15—C10—C11—C12	−1.2 (6)	C30—C25—C26—C27	1.2 (6)
C9—C10—C11—C12	177.8 (4)	C24—C25—C26—C27	−174.9 (4)
C10—C11—C12—C13	0.6 (7)	C25—C26—C27—C28	0.2 (6)
C11—C12—C13—C14	0.2 (7)	C26—C27—C28—C29	−2.1 (6)
C12—C13—C14—C15	−0.3 (7)	C27—C28—C29—C30	2.6 (7)
C13—C14—C15—C10	−0.3 (6)	C26—C25—C30—C29	−0.7 (7)
C11—C10—C15—C14	1.0 (6)	C24—C25—C30—C29	175.5 (4)
C9—C10—C15—C14	−177.9 (4)	C28—C29—C30—C25	−1.2 (7)
